# SIGNAL DETECTION AND VALIDATION IN AN ERA OF BIG GERONTOLOGICAL DATA

**DOI:** 10.1093/geroni/igac059.712

**Published:** 2022-12-20

**Authors:** Karen Bandeen-Roche

**Affiliations:** Johns Hopkins Bloomberg School of Public Health, Baltimore, Maryland, United States

## Abstract

Older adult health assessment long has posed measurement challenges—multidimensionality of sentinel outcomes like functioning and frailty, for example. This presentation discusses three developments creating opportunities for gerontologic biostatistics (GBS) over the past 10 years. Firstly, modeling to internally validate measurements or to quantify systematic heterogeneity in assessing older adult health has become considerably more widespread. Confirmatory latent variable modeling, harmonization, and mixture models will be addressed. Secondly, signal intensive behavioral phenotypes are proliferating, e.g. accelerometry, sleep actigraphy, and ecological momentary assessment. Functional data analysis is described as a data analytic technique to extract signal capturing main behavioral features or most relevant for health outcomes. Thirdly, “deep” characterization is under hot pursuit—whether by single- or multi-‘omics studies, or by multi-modal phenotyping as is increasingly common in the study of cognition. Techniques to accomplish this replicably are discussed. Throughout, potential pitfalls and implications for gerontologic data science development are identified.

